# The determinants of female genital mutilation among daughters in Nigeria

**DOI:** 10.1371/journal.pgph.0004413

**Published:** 2025-04-07

**Authors:** Charles Echezona Nzelu, Uche Maureen Nzelu, Amara Rita Ugwunze, Ngozi Azodoh

**Affiliations:** 1 Department of Special Projects, Federal Ministry of Health Nigeria, Federal Capital Territory, Nigeria; 2 National Examinations Council, Minna, Nigeria; 3 Department of Health Planning, Research and Statistics, Federal Ministry of Health Nigeria, Federal Capital Territory, Nigeria; University of New South Wales - Kensington Campus: University of New South Wales, AUSTRALIA

## Abstract

Female Genital Mutilation/Circumcision (FGM/C) refers to the complete or incomplete removal of the female external genital or other injuries for non-medical or cultural reasons. It is a culturally harmful practice without health benefits with long-term complications to women’s psychological, physical, and sexual health and well-being that violates the victims’ rights because it is done without their consent. Despite International efforts to eradicate this harmful practice, it persists, therefore, this study examined the sociodemographic factors of mothers of reproductive age that affected the likelihood of their daughters undergoing female genital mutilation or circumcision. The 2021 Multiple Indicator Cluster Survey (MICS) female genital mutilation response weighted data of women of reproductive age 15-49 years was used for this study. Bivariate and multivariable logistic regression analyses were done to predict the determinants of female genital mutilation among daughters, and those with a p-value of ≤.05 were considered statistically significant determinants. Results showed a statistically significant difference between educational level, ethnicity of household head, household wealth index, geopolitical zone, ever circumcised, and daughter circumcised at Alpha =.05 in the multivariable regression analysis. Place of Residence and Marital Status were not statistically significant in the multivariable regression analysis at Alpha =.05. Considering that Nigeria has a population of over 200 million people, a figure of 14.2% of daughters circumcised is high and, therefore, requires concerted interventions by all stakeholders to address this harmful practice.

## Introduction

According to a joint World Health Organization(WHO)/United Nations Children Fund (UNICEF)/United Nations Fund for Population Activities (UNFPA) statement, female genital mutilation(FGM) is defined as the complete or incomplete removal of the female external genital or other injuries to it for non-medical or cultural reasons [1] These organizations jointly classified Type I of FGM as the removal of the foreskin of the clitoris with or without the removal of part or all of the clitoris [1]. Type II is classified as the removal of the clitoris with incomplete or complete removal of the labia minora [1]. Type III FGM is the partial or total removal of the external genitalia and sewing/reducing the size of the vaginal opening, otherwise known as infibulation [1]. Type IV FGM involves all other injurious procedures to the female genitalia for cultural and non-therapeutic reasons, such as cutting, piercing or puncturing the clitoris or the labia or both, scrapping, and burning of the clitoris [1].

FGM is a culturally harmful practice with long-term effect on the women’s psychological, physical, and sexual health well-being [1–4]. The pain of undergoing this this procedure without anaesthesia, the anxiety, bleeding, likely resultant infection if the procedure is not sterile, reduction in libido and narrowing of the birth canal which may affect child delivery in the future underscore the importance of ending this non-beneficial practice [5,6]. It violates the victims’ rights [1–4]. Some of the cultural factors driving female genital mutilation in Nigeria include improved family social status and honour, initiation rite into womanhood, promotion of women’s cleanliness and hygiene, the better look of the female genitalia, improved marriageability prospects and control of female sexual desire [7–9].

The Nigerian Government in 2015 passed the Violence Against Persons Prohibition Act banning FGM and other harmful traditional practices [10]. The World Health Organization estimates that more than 200 million women and girls globally are affected by female genital mutilation/circumcision, and every year, 3 million girls are at risk of undergoing the procedure before the age of 15 years [2,11]. UNFPA estimates that between 2015 and 2030, 68 million girls will be at risk of female genital mutilation globally [12]. The World Health Assembly passed a resolution in 2008 (WHA61.16) on the elimination of FGM, stressing the importance of united actions in all sectors against FGM [13]. In 2012, the United Nations General Assembly adopted a resolution on the elimination of female genital mutilation [14]. Despite these resolutions, the practice of FGM remains high in West and Central Africa, with both accounting for 17 out of 27 countries in Africa where FGM is prevalent, with Nigeria accounting for 22 per cent of the 68 million women and girls at risk of FGM globally by 2030 [15]. According to the 2018 Nigeria Demographic and Health Survey, 19.2% of daughters have been circumcised in Nigeria, while 20% of women aged 15-49 years also in Nigeria have undergone female genital mutilation, with type 2 being the most common [16]. The results of 2013 and 2018 NDHS showed that the rate of FGM in Nigeria decreased from 25% in 2013 to 20% in 2018. Tribe, religion, geopolitical zone, educational level, residence, age, and household wealth quintile are associated with the likelihood of women and their daughters undergoing FGM [16,17]. Igwegbe and Egbonu, in a Nigerian study, reported that the level of education significantly impacted the likelihood of undergoing FGM [18]. The results of a study done on “Socio-economic and demographic determinants of female genital mutilation in sub-Saharan Africa: analysis of data from demographic and health surveys” showed that FGM among women and their daughters who are in the richest wealth quintile, with higher levels of education are less likely to undergo FGM compared to those in the poorest wealth quintile, and lower levels of education respectively [19]. Also, FGM among women and their daughters increased with age and marital status; however, while women in rural areas were less likely to undergo FGM, their daughters were more likely to undergo FGM [19]. Many studies have reported that the circumcision status of mothers, educational level, age categories of mothers, place of residence, household wealth quintile, ethnicity, religion and marital status impact the likelihood of women or their daughters undergoing FGM [16–21]. With the availability of newer datasets on FGM [23], this study examined the sociodemographic factors of mothers of reproductive age that affected the likelihood of their daughters undergoing female genital mutilation or circumcision. It will assist in identifying determinants still impacting female genital mutilation in Nigeria in the face of the reported reduction in its practice between 2013 and 2018 [16,17]. and the development of interventions to address its occurrence. Sociodemographic variables identified in previous studies as determinants of female genital mutilation, which data were collected in the primary study, were included in this study as independent/predictor variables. The identified variables include educational level, age categories, marital status, place of residence, ever circumcised by mother, geopolitical zone, ethnicity of head of household and wealth index

## Materials and methods

### Data source and design

The 2021 Multiple Indicator Cluster Survey (MICS) Women of reproductive age 15-49 years female genital mutilation responses weighted data was used for this study. The study is cross-sectional, nationally representative and used a multi-stage stratified cluster design to select the study participants. The Survey was designed to provide multiple indicators estimates for women and children at the national level, 36 states of Nigeria, urban and rural areas, the Federal Capital Territory (FCT) Abuja and the country’s Six Geo-political zones. The total sample size targeted for the survey was 1,850 clusters and 37,000 households. Women of reproductive age 15-49, Men 15-49 years and Children 0-17 years were included in the survey. Data on the variables of interest was obtained using the women’s questionnaire (see S1 Data). An in-depth description of the methodology of the 2021 MICS has been provided elsewhere [22].

### Variable measures

The dependent variable is the dichotomus variable “Daughter Circumcized” which refers to the girl children between the ages of 0-14 years and coded as Circumcised = 0 and Not Circumcised = 1. The independent variables were Marital Status recoded as Ever Married = 1 and Never Married = 2; Educational level was recoded as No Education = 0, Primary Education =1, Secondary Education = 2, Tertiary Education = 3. Place of Residence coded as Urban = 1, Rural = 2. The Geopolitical Zone was coded as North-Central = 1, North-East = 2, and North-West = 3. South-East = 4, South-South = 5 and South-West = 6. Household Wealth Index is a cumulative household living standard composite measure calculated using the data collected on household ownership of assets such as bicycles, house construction materials, television, sanitation facilities, and water access type. Based on the principal component analysis result, it was graded as poorest, second, middle, fourth and richest. For this study, the Wealth Index was recoded as Poor Household Wealth Index = 1 (combining poorest and second), Average Household Wealth Index = 2 (middle) and Rich Household Wealth Index = 3 (combining fourth and richest). Ever Circumcised by Mother coded as Yes = 1, No = 2, The age of the women was categorised in ascending order into five-year interval groups (15-19, 20-24, 25-29, 30-34, 35-39, 40-44, 45-49). The ethnicity of the household head was coded as Hausa = 1, Igbo = 2, Yoruba = 3, Fulani = 4, Kanuri = 5, Ijaw = 6, Tiv = 7, Ibibio = 8, Edo = 9, Other Ethnicity = 96. The Don’t Know and No Response categories were treated as user-defined missing data. The determinant variables were measured on the nominal scale except Age Categories, which were measured on an interval scale to facilitate multiple imputations.

### Data management and analysis

The Statistical Package for Social Sciences (SPSS) Version 29 was used for the study analysis. The level of significance was set at Alpha =.05. Descriptive statistics of the study variables showed that some of them had missing data. Little’s MCAR test was conducted to assess if the data was missing completely at random. The result showed that it was not, and therefore, multiple imputations using the regression method were done to handle selection bias resulting from the non-randomness of the missing data [23,24]. To ensure that the sample is representative of the target population, the non-proportional allocation of samples to states and places of residence, including the different response rates in the primary study, was addressed by applying the sample weight provided by the implementers of the Multiple Indicator Cluster Survey (MICS) in the dataset [22]. Bivariable and multivariable logistic regression analyses predicted the relationship between these determinant variables and daughter circumcised. Determinants not statistically significant at Alpha =.05 were not included in the multivariable regression analysis. Crude and adjusted odds ratios with 95% confidence interval were reported for different categories of the predictor variables relative to their reference categories. Cross-tabulation between the determinants and the dependent variable was used to get the valid weighted frequencies and percentages.

### Ethical procedures

This study is a secondary analysis of publicly available de-identified data from the 2021 Multiple Indicator Cluster Survey; therefore, anonymity was maintained. We requested permission to use the dataset, which was granted to download the data for the study from https://mics.unicef.org/surveys (see S1 Text). The procedure approved by the MICS public-use datasets does not permit respondents, households, or sample communities to be identified. The data files do not contain personal identifiers of individuals or households.

## Results

Table 1 shows the frequency distribution of the determinant variables and the dependent variable (educational level, age categories, marital status, place of residence, ever circumcised, geopolitical zone, ethnicity of head of household, wealth index and daughter circumcised) of the study participants. The total un-weighted studies sample size is 24836 while the total weighted studied sample size is 26233.

**Table 1:** Frequency distribution of the sociodemographic characteristics of the women of reproductive age 15-49 years in the study.

Predictors	Weighted Frequency (n)	Weighted Percent (%)
**Educational Level**		
No Education	6642	25.3
Primary Education	5408	20.6
Secondary	10350	39.5
Tertiary	3833	14.6
**Age Categories (Years)**		
15–19	6856	43.0
20–24	5280	33.1
25–29	2830	17.8
30–34	758	4.8
35–39	179	1.1
40–44	26	0.2
45–49	2	0.0
**Place of Residence**		
Urban	13637	51.98
Rural	12596	48.02
**Marital Status**		
Ever Married	25956	98.9
Never Married	277	1.1
**Household Wealth Index**		
Poor Household Wealth Index	7721	29.4
Average Household Wealth Index	5557	21.2
Rich Household Wealth Index	12955	49.4
**Ethnicity of Household Head**		
Hausa	5288	20.2
Igbo	4966	18.9
Yoruba	5873	22.4
Fulani	1419	5.4
Kanuri	622	2.4
Ijaw	736	2.8
Tiv	469	1.8
Ibibio	645	2.5
Edo	582	2.2
Other Ethnicity	5633	21.5
**Geopolitical Zone**		
North Central	2902	11.1
North East	2721	10.4
North West	5075	19.3
South East	3887	14.8
South South	4978	19.0
South West	6670	25.4
**Ever Circumcised by Mother**		
Yes	9039	34.46
No	17194	64.54
**Daughter Circumcised**		
Circumcised	3788	14.4
Not Circumcised	22445	85.6

**Fig 1:**
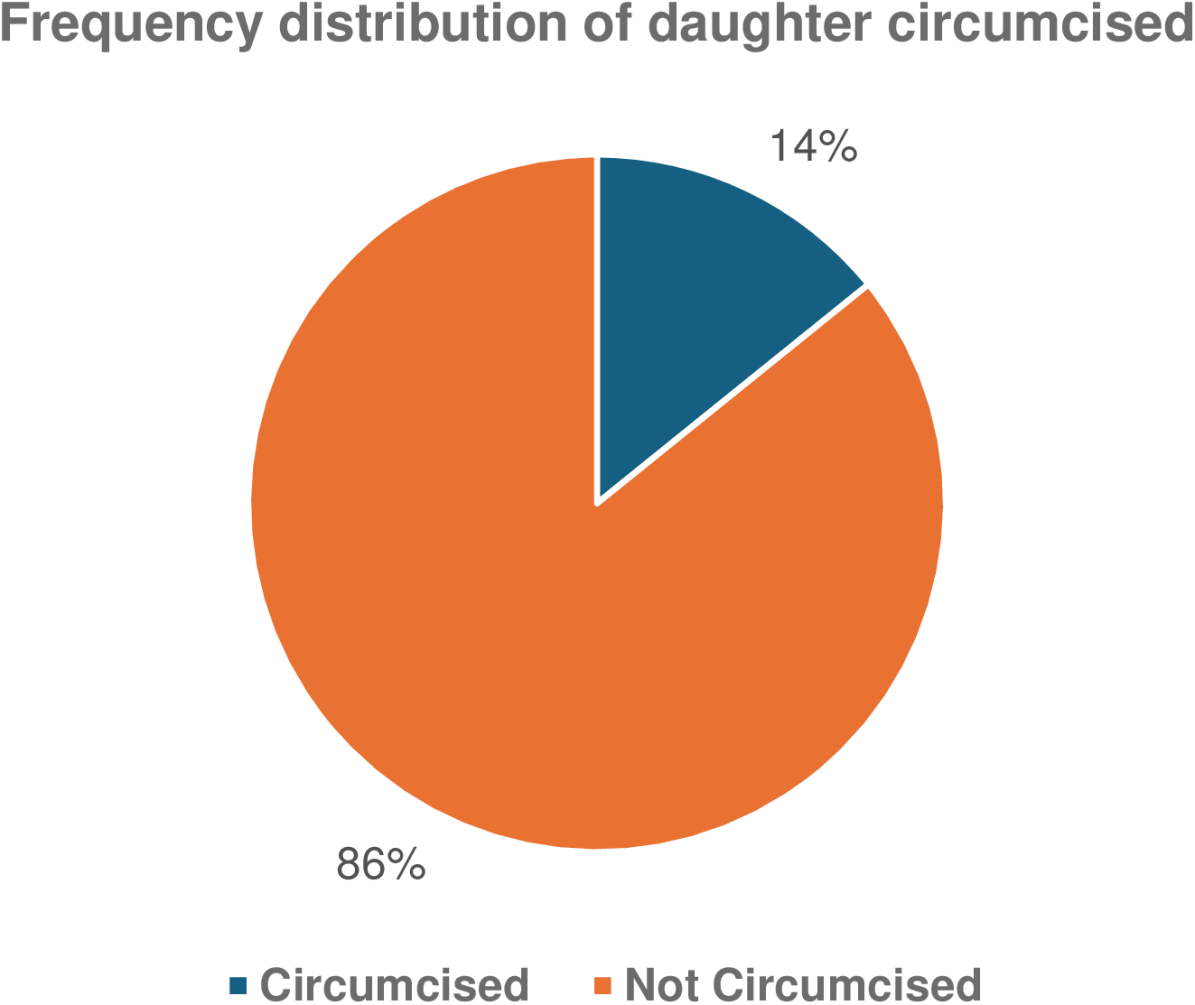
Frequency distribution of daughters circumcised.

Women of reproductive age with secondary and no education were highest in the sample, with 10350 (39.5%) and 6642 (25.3%) respectively, followed by those with primary education 5408(20.6%) and tertiary education 3833(16.4%). Ever-married women were more in the sample 25956 (98.1%) than those never married 277 (1.1%) and most women lived in urban areas. Younger women aged 15-19 were more in the study, while those between 45-49 years were the least. The practice of FGM ranges from 10.4% in the North-Central zone to 25.4% in the South-West zone.  Fig 1 shows that the number of daughters circumcised was 3788 (14.4%), while those not circumcised were 22445 (85.6%).

Most of the daughters in this study were not circumcised.

The crude and adjusted odds ratios from bivariable and multivariable logistic regression analyses are presented in Table 2. There was a statistically significant difference between Educational Level, ethnicity of household head, household wealth index, geopolitical zone, ever circumcised, and daughter circumcised at Alpha =.05 in the multivariable regression analysis. Marital Status was not statistically significant in the multivariable regression analysis at Alpha =.05. Although age categories were statistically significant in the bivariable regression analysis, it was omitted in the multivariable regression analysis because a category (ages 45-49) contained small sample size that gave an unreliable estimate. Place of Residence was not statistically significant in the bivariable regression analysis and therefore was omitted from the multivariable regression analysis. Women of reproductive age with Tertiary Levels of Education were 2.15 times more statistically significantly likely not to have their daughters circumcised than those with no education (AOR = 2.15: 95% CI, 1.60, 2.90). There was no difference in the practice of female genital mutilation between women with primary and secondary education and those with no education. Women with an Average Household Wealth Index (AOR =.87: 95% CI;.75,1.02) and those with a Rich Household Wealth Index (AOR =1.17: 95% CI;.98, 1.38) were not statistically significantly likely not to have their daughters circumcised than those with Poor Household Wealth Index. The practice of female genital mutilation among daughters is expected to be less common among women with higher levels of household wealth index [23]. This is because they are expected to be more educated and have more access to the mass media where information influencing their practice of female genital mutilation. Women who were never circumcised were 58.53 times (95% CI; 37.81, 90.61) more statistically significantly likely not to have their daughter circumcised than those who have been circumcised. Women from the North-West geopolitical zone were statistically significantly less likely to have their daughters not circumcised than those from the North-Central geopolitical zone (AOR =.12: 95% CI;.08,.17). Women or mothers from the other geopolitical zones were more likely to have their daughters not circumcised than those from the North-Central zone. Women whose heads of their households’ ethnicity were from the Igbo (AOR = 1.67: 95% CI; 1.08, 2.57), Ijaw, Tiv, Ibibio, and Other Ethnicities are more statistically significantly likely not to have their daughters circumcised than those from the Hausa ethnic group at Alpha =.05. Women whose heads of households were from the Yoruba (AOR =.49: 95% CI;.35,.70) and Fulani, were statistically significantly less likely to have daughters e not circumcised} than those from the Hausa ethnic group at Alpha =.05. There was no difference in the practice of FGM among the Hausa, Kanuri and Edo ethnic groups.

**Table 2:** Logistic regression models of the determinants of female genital mutilation in Nigeria.

Predictors	COR (95% C.I.), *p*-value	AOR (95% C.I.), *p*-value
**Educational Level**	*p* <.001	
No Education	Ref	*Ref*
Primary Education	1.43 (1.27, 1.61); *p* <.001	.97 (.74, 1.26); *p =*.78
Secondary	2.40 (2.08, 2.78); *p* <.001	1.25 (.93, 1.69); *p* =.13
Tertiary	4.96 (4.04, 6.09); *p* <.001	2.15 (1.60, 2.91); *p <.001*
**Age Categories (Years)**	*p* <.001	
15-19	Ref	
20 – 24	1.25 (1.13, 1.39); *p = *<.001	–
25 – 29	1.93 (1.66, 2.24); *p = *<.001	–
30 – 34	1.37 (1.08, 1.73); *p* =.008	–
35 – 39	2.53 (1.39, 4.60); *p* =.002	–
40 – 44	2.40 (.52, 11.08); *p* =.26	–
45 - 49	284608383.91(.000,-) *p* =.10	–
**Ever Circumcised**		
Yes	Ref	Ref
No	1.81(1.69, 1.93); *p =.001*	58.5(37.81, 90.61) p <.001
**Place of Residence**		
Urban	Ref	Ref
Rural	.94 (.83,.1.06); *p = *.27	–
**Marital Status**		
Ever Married	Ref	Ref
Never Married	3.92 (2.01, 7.61, *p* <.001	1.78 (.70, 4.58); p =.22
**Household Wealth Index**	*p* <.001	
Poor Household Wealth Index	Ref	Ref
Average Household Wealth Index	1.32 (1.19, 1.47); *p = *<.001	.87 (.75, 1.02); p =.09
Rich Household Wealth Index	1.95 (1.73, 2.17); *p* <.001	1.17 (.98, 1.38); p =.08
**Geopolitical Zone**	p <.001	
North-Central	Ref:	*Ref*
North-East	8.37 (5.74, 12.23); p <.001	2.28 (1.51, 3.45); p <.001
North-West	.24 (.21,.27); p = <.001	.13 (.09,.17); p <.001
South-East	2.92(2.18; 3.92); p <.001	3.15 (2.22, 4.47); p <.001
South-South	2.02 (1.65, 2.47), p <.001	1.65 (1.18, 2.33); p =.006
South-West	.87 (.76,.1.00); p =.06	3.31 (2.58, 4.25); p <.001
**Ethnicity of Household Head**	p <.001	
Hausa	*Ref*	*Ref*
Igbo	6.93(5.57, 8.63); p <.001	2.09(1.1.41, 3.12); p <.001
Yoruba	1.50 (1.35, 1.67); p <.001	.64 (.48,.86); p <.003
Fulani	.81 (.71,.93); p <.001	.43 (.34,.54); p <.001
Kanuri	4.79(2.92, 7.86); p <.001	2.02 (.55, 7.39), p =.24
Ijaw	288.03 (33.06, 2509.52); p <.001	166.27 (18.78, 1472.02); p <.001
Tiv	102.56 (16.47, 638.85);p <.001	9.80 (1.46, 65.93); p =.02
Ibibio	15.26 (8.69, 26.78); p <.001	5.33 (2.75, 10.32); p <.001
Edo	3.21 (2.23, 4.61); p <.001	1.57(.93 2.65); p =.09
Other Ethnicity	6.60 (5.44, 8.400); p <.001	3.34 (2.31, 4.81); p <.001

## Discussion

The Multivariable Logistic Regression Analysis results in this study showed that Educational Level, Ever Circumcised, Geopolitical Zone, and Ethnicity of Head of Household, statistically significantly impact female genital mutilation of daughters at Alpha =.05. Marital status, household wealth index and place of residence werenot statistically significantly associated with female genital mutilation of daughters.

This study reported that women with higher educational levels are less likely to have their daughters circumcised than those with no education and lower levels of education. Igwebe and Egbonu, Ahinkorah, Ahinkorah et al., corroborated this finding in their studies in Nigeria, Chad and Sub-Saharan Africa respectively [18,19,25]. This is not surprising as higher levels of education have been considered an essential tool for changing individual’s attitudes towards many negative socio-cultural practices including female genital mutilation [26]. Also, higher levels of education provide women with some levels of empowerment to refuse negative socio-cultural practices where societal pressure compels them [27]. This study found that women who have never been circumcised are more likely not to have their daughters circumcised than those who have been circumcised. It is a continuation of what the 2018 Nigeria Demographic and Health Survey reported about female genital mutilation [16]. This is consistent with what Obi and Igbinadolor found in their study in Benin City, Edo State Nigeria which also reported that ever-circumcised mothers have more intention to circumcise their daughters in the future [20]. Also, many other studies supported this finding that ever-circumcised women are more likely to circumcise their daughters [19,25,28]. Ever-circumcised mothers are more likely to circumcise their daughters because of the societal norms handed down to them by their mothers and grandmothers, and its discontinuation is met with societal pressure and the risk of isolation [29]. Apart from women who are from the North-West Geopolitical zone in Nigeria who were more likely to have their daughters circumcised than those from the North-Central zone, other women from the other four Geopolitical zones were less likely to have their daughters circumcised than those from the North-Central zone. These variations in the practice of FGM across the six geopolitical zones in Nigeria may be related to the effects of socio-cultural determinants of FGM such as the women’s educational level, ever-circumcised, household wealth index, cultural and religious practices prevalent in these different geopolitical zones [20,30,31]. Women whose heads of their households’ ethnicity were from the Igbo, Ijaw, Tiv, Ibibio, and Other Ethnicities are more likely to have their daughters not circumcised than those from the Hausa ethnic group at Alpha =.05. This finding is supported by other studies that found different prevalences of female genital mutilation among different ethnic groups in Nigeria [20]. Different ethnic groups in Nigeria and Africa practice female genital mutilation due to various sociocultural ideas and reasons such as chastity, promiscuity, purity, fertility, hygiene, religion and cultural identity [18,20,30,32–34]. These sociocultural ideas and reasons have perpetuated and sustained the practice of FGM of daughters among the various ethnic groups. The adjusted odd ratio for the Ijaw ethnic group was high and it may be due to outlier’s value in the dataset. The individual categories of the Household Wealth Index of the mothers did not impact the circumcision of their daughters in this study. This is consistent with Ahinkorah’s findings in a study done in Chad [25]. Although Household Wealth Index individual categories in this study did not statistically significantly impact female genital mutilation, many other studies reported that women with higher household wealth index are more likely not to have their children circumcised than those with lower levels of household wealth index [19,31,34].

## Limitations

A limitation of this study is recall bias which may have impacted the reliability and validity of this study’s findings because the Multiple Indicator Cluster Survey was a self-report of the study participants. Also, unmeasured confounding impacted the study’s findings because the study variables were restricted to the ones collected in the 2021 MICS.

## Conclusion

This study found that Educational Level, Ever Circumcised, Geopolitical Zone, Ethnicity of Head of Household, and Household Wealth Index statistically significantly impacted female genital mutilation. The implication is that if these identified determinants of FGM are not tackled, the practice of FGM will remain high in Nigeria. Therefore, the identification of these determinants of female genital mutilation will assist the government and other relevant stakeholders in designing informed policies, strategies, and interventions for the reduction and, ultimately, the stoppage of female genital mutilation in Nigeria. Also, this study’s findings identified the determinants that are associated with FGM in Nigeria and addressing them will assist in reducing the prevalence both in Nigeria and globally. Public health education on the danger or consequences of the continuation of the FGM of girl children in Nigeria should be intensified. The government of Nigeria having passed a law against FGM should consider strict enforcement of the law to discourage the continuation of this harmful practice.

## Supporting information

S1 Text Authorization letter by UNICEF to use MICS dataset. (PDF)

S1 Data SPSS dataset for the study “Determinants of Female Genital Mutilation among daughters in Nigeria”. (SAV)
