# Supplementary material for: The determinants of female genital mutilation among daughters in Nigeria
Source: PLOS Glob Public Health. 2025 Apr 7;5(4):e0004413. doi: 10.1371/journal.pgph.0004413 (PMC11975082; doi:10.1371/journal.pgph.0004413)
Supplement: S1 Text — (PDF) [file pgph.0004413.s001.pdf]

HOME MAIL NEWS FINANCE SPORT CELEBRITY STYLE WEATHER MORE... y!mail+ Upgrade now

yahoo!mail Find messages, documents, photos or people Advanced Home

Compose

charlesnzelu@ya... 68K
charlesnzelu 23K

Inbox 68K
Unread
Starred
Drafts 856
Sent
Archive
Spam
Deleted Items
Less

Views Hide
Photos
Documents
Subscriptions
Travel

Folders Show

Back Archive Move Delete Sp

Access to MICS datasets approved

Yahoo/Inbox

no-reply@unicef.org Tue, 4 Jun at 21:03

From:
no-reply@unicef.org
To:
charlesnzelu@yahoo.co.uk

Dear Charles,

Thank you for requesting to use the MICS dataset(s). You have been granted access.

The data may not be used for purposes other than those expressed in the application form and may not be redistributed or passed on to others in any form.

Please log into the MICS website using the email and password you provided during registration and download the dataset(s) on survey page.

We would appreciate if you would share your research findings with us – please email us at mics@unicef.org.

Best regards,

UNICEF MICS Team

Reply, Reply all or Forward
